# (*E*)-4-Allyl-2-[(2-hydroxy­phen­yl)iminiometh­yl]-6-methoxy­phenolate

**DOI:** 10.1107/S160053681000838X

**Published:** 2010-03-27

**Authors:** Naser Eltaher Eltayeb, Siang Guan Teoh, Suchada Chantrapromma, Hoong-Kun Fun

**Affiliations:** a School of Chemical Sciences, Universiti Sains Malaysia, 11800 USM, Penang, Malaysia; b Crystal Materials Research Unit, Department of Chemistry, Faculty of Science, Prince of Songkla University, Hat-Yai, Songkhla 90112, Thailand; c X-ray Crystallography Unit, School of Physics, Universiti Sains Malaysia, 11800 USM, Penang, Malaysia

## Abstract

The title compound, C_17_H_17_NO_3_, crystallizes in a zwitterionic form with cationic iminium and anionic enolate groups. The zwitterion exists in a *trans* configuration about the C=N bond. The dihedral angle between the two benzene rings is 13.42 (7)°. The meth­oxy group is almost coplanar [C—O—C—C = 2.1 (2)°] with the attached ring whereas the allyl unit is oriented at a dihedral angle of 67.9 (1)°. An intra­molecular N—H⋯O hydrogen bond generates an *S*(6) ring motif. In the crystal, the mol­ecules are linked into zigzag chains along [010] by O—H⋯O hydrogen bonds. In addition, weak C—H⋯π inter­actions are observed.

## Related literature

For background to Schiff bases and their applications, see: Dao *et al.* (2000 ▶); Eltayeb & Ahmed (2005*a* ▶,*b* ▶); Karthikeyan *et al.* (2006 ▶); Sriram *et al.* (2006 ▶). For related structures, see: Eltayeb *et al.* (2009 ▶); Tan & Liu (2009 ▶). For bond-length data, see: Allen *et al.* (1987 ▶). For hydrogen-bond motifs, see: Bernstein *et al.* (1995 ▶). For the stability of the temperature controller used in the data collection, see: Cosier & Glazer (1986 ▶).
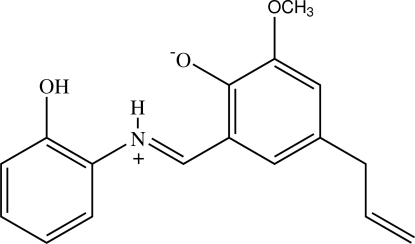


## Experimental

### 

#### Crystal data

C_17_H_17_NO_3_
*M* *_r_* = 283.32
Orthorhombic, 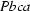

*a* = 14.719 (3) Å
*b* = 9.1302 (16) Å
*c* = 20.597 (4) Å
*V* = 2768.0 (9) Å^3^
*Z* = 8
Mo *K*α radiation
μ = 0.09 mm^−1^
*T* = 100 K
0.50 × 0.15 × 0.02 mm

#### Data collection

Bruker APEX DUO CCD area-detector diffractometer
Absorption correction: multi-scan (*SADABS*; Bruker, 2009 ▶) *T*_min_ = 0.955, *T*_max_ = 0.998
16563 measured reflections
4056 independent reflections
2744 reflections with *I* > 2σ(*I*)
*R*_int_ = 0.073

#### Refinement

*R*[*F* ^2^ > 2σ(*F* ^2^)] = 0.052
*wR*(*F* ^2^) = 0.141
*S* = 1.00
4056 reflections
254 parameters
H atoms treated by a mixture of independent and constrained refinement
Δρ_max_ = 0.39 e Å^−3^
Δρ_min_ = −0.29 e Å^−3^

### 

Data collection: *APEX2* (Bruker, 2009 ▶); cell refinement: *SAINT* (Bruker, 2009 ▶); data reduction: *SAINT*; program(s) used to solve structure: *SHELXTL* (Sheldrick, 2008 ▶); program(s) used to refine structure: *SHELXTL*; molecular graphics: *SHELXTL*; software used to prepare material for publication: *SHELXTL* and *PLATON* (Spek, 2009 ▶).

## Supplementary Material

Crystal structure: contains datablocks global, I. DOI: 10.1107/S160053681000838X/ci5052sup1.cif

Structure factors: contains datablocks I. DOI: 10.1107/S160053681000838X/ci5052Isup2.hkl

Additional supplementary materials:  crystallographic information; 3D view; checkCIF report

## Figures and Tables

**Table 1:** Hydrogen-bond geometry (Å, °) *Cg*1 is the centroid of the C8–C13 ring.

*D*—H⋯*A*	*D*—H	H⋯*A*	*D*⋯*A*	*D*—H⋯*A*
O1—H1*O*1⋯O2^i^	0.95	1.72	2.6443 (16)	166
O1—H1*O*1⋯O3^i^	0.95	2.55	3.1268 (16)	119
N1—H1*N*1⋯O2	0.97 (2)	1.85 (2)	2.6553 (18)	138 (2)
C14—H14*B*⋯*Cg*1^ii^	1.01 (2)	2.75 (2)	3.569 (2)	139 (2)

Symmetry codes: (i) 
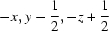
; (ii) 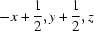.

**Fig. 1.**
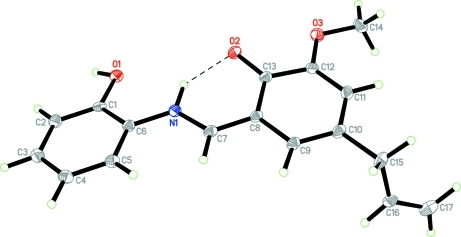
The molecular structure of the title compound, with 50% probability displacement ellipsoids and the atom-numbering scheme. The dashed line indicates a hydrogen bond.

**Fig. 2.**
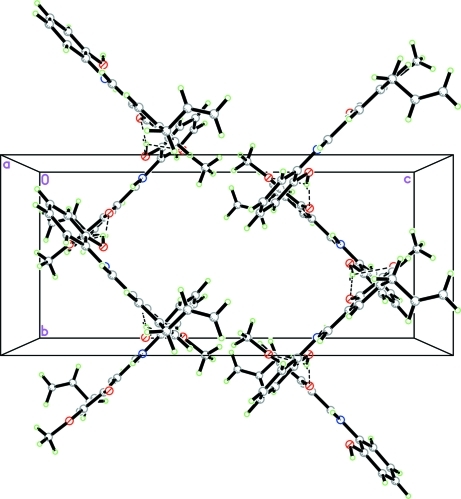
The crystal packing of the title compound, viewed down the a axis, showing chains running along the b axis. Hydrogen bonds are shown as dashed lines.

## supplementary crystallographic information

### Comment

Schiff bases have received much attention because of their potential applications. Some of these compounds exhibit various pharmacological activities, as noted by their anticancer (Dao *et al.*, 2000), anti-HIV (Sriram *et al.*, 2006), antibacterial and antifungal (Karthikeyan *et al.*, 2006) properties. In addition, some of them may be used as analytical reagents for the determination of trace elements (Eltayeb & Ahmed, 2005*a*,*b*). Previously we have reported the crystal structure of 2-((*E*)-{2-[(*E*)-2,3-dihydroxybenzylideneamino]-5-methylphenyl}- iminiomethyl)-6-hydroxyphenolate (Eltayeb *et al.*, 2009) which exists in a zwitterionic form. The title compound is another schiff base which also crystallizes in a zwitterionic form. Herein we report its crystal structure.

The title molecule exists in a *trans* configuration about the C═N double  bond [1.3129 (19) Å], with a C6–N1–C7–C8 torsion angle of 178.13 (14)°. The molecule is slightly twisted, with the dihedral angle between the two benzene rings being 13.42 (7)°. The hydroxy group is coplanar with the attached C1–C6 benzene ring. The methoxy group is coplanar with the attached C8–C13 benzene ring [C14—O3—C12—C11 = 2.1 (2)°; r.m.s. deviation of 0.0384 (1) Å for the eight non H atoms]. The allyl unit (C15–C17) is in an (-)-anticlinal conformation as indicated by the C11—C10—C15—C16 torsion angle of -94.28 (18)°. An intramolecular N—H···O hydrogen bond between the NH^+^ and the phenolate O^-^ groups generate an S(6) ring motif (Fig. 1 and Table 1) (Bernstein *et al.*, 1995). The bond distances show normal values (Allen *et al.*, 1987) and are comparable with those observed in related structures (Eltayeb *et al.*, 2009; Tan & Liu, 2009).

In the crystal packing (Fig. 2), molecules are linked into zigzag chains along the [010] by O—H···O hydrogen bonds involving hydroxy groups, phenolate and methoxy O atoms (Table 1). Within a chain, the adjacent molecules are approximately perpendicular to each other (Fig. 2). The crystal structure is further stabilized by weak intermolecular C—H···π interactions (Table 1) involving the C8–C13 ring (centroid Cg1).

### Experimental

The title compound was synthesized by adding 5-allyl-2-hydroxy-3-methoxybenzaldehyde (0.768 g, 4 mmol) to a solution of 2-aminophenol (0.436 g, 4 mmol) in ethanol (30 ml). The mixture was refluxed with stirring for 30 min. The resultant red solution was filtered and the filtrate was evaporated to give a red solid product. Red plate-shaped single crystals of the title compound suitable for X-ray structure determination were obtained from diethyl ether by slow evaporation at room temperature after a few days.

### Refinement

Atom H1O1 attached to O1 was located in a difference map and then constrained to ride with U_iso_ = 1.5*U*_eq_(O1). The remaining H atoms were also located in a difference map and were isotropically refined.

### Crystal data

**Table:**

C_17_H_17_NO_3_	*F*(000) = 1200
*M**_r_* = 283.32	*D*_x_ = 1.360 Mg m^−^^3^
Orthorhombic, *P**b**c**a*	Mo *K*α radiation, λ = 0.71073 Å
Hall symbol: -P 2ac 2ab	Cell parameters from 4056 reflections
*a* = 14.719 (3) Å	θ = 2.0–30.2°
*b* = 9.1302 (16) Å	µ = 0.09 mm^−^^1^
*c* = 20.597 (4) Å	*T* = 100 K
*V* = 2768.0 (9) Å^3^	Plate, red
*Z* = 8	0.50 × 0.15 × 0.02 mm

### Data collection

**Table:**

Bruker APEX DUO CCD area-detector diffractometer	4056 independent reflections
Radiation source: sealed tube	2744 reflections with *I* > 2σ(*I*)
graphite	*R*_int_ = 0.073
φ and ω scans	θ_max_ = 30.2°, θ_min_ = 2.0°
Absorption correction: multi-scan (*SADABS*; Bruker, 2009)	*h* = −20→20
*T*_min_ = 0.955, *T*_max_ = 0.998	*k* = −12→12
16563 measured reflections	*l* = −26→28

### Refinement

**Table:**

Refinement on *F*^2^	Primary atom site location: structure-invariant direct methods
Least-squares matrix: full	Secondary atom site location: difference Fourier map
*R*[*F*^2^ > 2σ(*F*^2^)] = 0.052	Hydrogen site location: inferred from neighbouring sites
*wR*(*F*^2^) = 0.141	H atoms treated by a mixture of independent and constrained refinement
*S* = 1.00	*w* = 1/[σ^2^(*F*_o_^2^) + (0.0776*P*)^2^] where *P* = (*F*_o_^2^ + 2*F*_c_^2^)/3
4056 reflections	(Δ/σ)_max_ = 0.001
254 parameters	Δρ_max_ = 0.39 e Å^−^^3^
0 restraints	Δρ_min_ = −0.29 e Å^−^^3^

### Special details

**Table:**

Experimental. The crystal was placed in the cold stream of an Oxford Cryosystems Cobra open-flow nitrogen cryostat (Cosier & Glazer, 1986) operating at 100.0 (1) K.
Geometry. All esds (except the esd in the dihedral angle between two l.s. planes) are estimated using the full covariance matrix. The cell esds are taken into account individually in the estimation of esds in distances, angles and torsion angles; correlations between esds in cell parameters are only used when they are defined by crystal symmetry. An approximate (isotropic) treatment of cell esds is used for estimating esds involving l.s. planes.
Refinement. Refinement of F^2^ against ALL reflections. The weighted R-factor wR and goodness of fit S are based on F^2^, conventional R-factors R are based on F, with F set to zero for negative F^2^. The threshold expression of F^2^ > 2sigma(F^2^) is used only for calculating R-factors(gt) etc. and is not relevant to the choice of reflections for refinement. R-factors based on F^2^ are statistically about twice as large as those based on F, and R- factors based on ALL data will be even larger.

### Fractional atomic coordinates and isotropic or equivalent isotropic displacement parameters (Å^2^)

**Table:**

	*x*	*y*	*z*	*U*_iso_*/*U*_eq_	
O1	0.01450 (7)	0.45448 (12)	0.30069 (5)	0.0161 (2)	
H1O1	−0.0369	0.3930	0.3044	0.024*	
O2	0.12030 (7)	0.76365 (12)	0.20618 (6)	0.0180 (3)	
O3	0.15126 (7)	0.93837 (13)	0.10464 (5)	0.0197 (3)	
N1	0.18381 (8)	0.56151 (14)	0.28699 (6)	0.0135 (3)	
C1	0.07946 (9)	0.39626 (16)	0.33947 (7)	0.0135 (3)	
C2	0.06178 (10)	0.28421 (18)	0.38347 (8)	0.0167 (3)	
C3	0.13112 (11)	0.22677 (18)	0.42119 (8)	0.0186 (3)	
C4	0.21898 (11)	0.28105 (18)	0.41570 (8)	0.0177 (3)	
C5	0.23708 (10)	0.39363 (17)	0.37262 (8)	0.0153 (3)	
C6	0.16809 (10)	0.45100 (16)	0.33395 (7)	0.0136 (3)	
C7	0.26375 (10)	0.60410 (17)	0.26593 (7)	0.0145 (3)	
C8	0.27799 (9)	0.71113 (16)	0.21797 (7)	0.0133 (3)	
C9	0.36954 (10)	0.74371 (17)	0.20005 (8)	0.0150 (3)	
C10	0.38828 (10)	0.84469 (17)	0.15316 (8)	0.0166 (3)	
C11	0.31474 (11)	0.91227 (17)	0.12006 (8)	0.0166 (3)	
C12	0.22584 (10)	0.88183 (16)	0.13544 (8)	0.0147 (3)	
C13	0.20324 (10)	0.78420 (16)	0.18785 (7)	0.0139 (3)	
C14	0.16825 (13)	1.03310 (19)	0.05078 (9)	0.0221 (4)	
C15	0.48556 (11)	0.8797 (2)	0.13422 (9)	0.0207 (4)	
C16	0.52342 (10)	0.77196 (19)	0.08627 (8)	0.0188 (3)	
C17	0.55487 (11)	0.8048 (2)	0.02828 (9)	0.0244 (4)	
H2	0.0021 (12)	0.247 (2)	0.3859 (8)	0.015 (4)*	
H3	0.1195 (13)	0.144 (2)	0.4528 (9)	0.025 (5)*	
H4	0.2678 (14)	0.238 (2)	0.4435 (10)	0.031 (5)*	
H5	0.2969 (13)	0.435 (2)	0.3707 (9)	0.023 (5)*	
H7	0.3152 (12)	0.5554 (19)	0.2862 (8)	0.015 (4)*	
H9	0.4179 (13)	0.6900 (19)	0.2254 (8)	0.019 (5)*	
H11	0.3273 (13)	0.983 (2)	0.0852 (9)	0.024 (5)*	
H14A	0.1109 (14)	1.062 (2)	0.0335 (10)	0.030 (5)*	
H14B	0.2011 (14)	1.124 (2)	0.0651 (10)	0.034 (6)*	
H14C	0.2072 (13)	0.985 (2)	0.0176 (9)	0.025 (5)*	
H15A	0.5199 (14)	0.882 (2)	0.1754 (10)	0.032 (6)*	
H15C	0.4901 (12)	0.982 (2)	0.1163 (9)	0.015 (4)*	
H16	0.5258 (13)	0.672 (2)	0.1013 (9)	0.029 (5)*	
H17A	0.5552 (14)	0.909 (2)	0.0120 (10)	0.033 (5)*	
H17B	0.5755 (15)	0.729 (2)	−0.0016 (10)	0.038 (6)*	
H1N1	0.1343 (15)	0.611 (2)	0.2647 (10)	0.035 (6)*	

### Atomic displacement parameters (Å^2^)

**Table:**

	*U*^11^	*U*^22^	*U*^33^	*U*^12^	*U*^13^	*U*^23^
O1	0.0080 (5)	0.0182 (6)	0.0222 (6)	−0.0005 (4)	−0.0012 (4)	0.0024 (5)
O2	0.0091 (5)	0.0203 (6)	0.0246 (6)	0.0008 (4)	0.0019 (4)	0.0038 (5)
O3	0.0153 (5)	0.0214 (6)	0.0223 (6)	0.0032 (4)	−0.0001 (4)	0.0063 (5)
N1	0.0101 (6)	0.0140 (6)	0.0164 (7)	−0.0008 (5)	−0.0003 (5)	0.0008 (5)
C1	0.0091 (6)	0.0140 (7)	0.0173 (8)	0.0023 (5)	−0.0004 (5)	−0.0023 (6)
C2	0.0127 (7)	0.0170 (8)	0.0204 (8)	−0.0020 (6)	0.0014 (6)	0.0011 (6)
C3	0.0170 (7)	0.0177 (8)	0.0211 (8)	0.0006 (6)	0.0012 (6)	0.0030 (6)
C4	0.0155 (7)	0.0180 (8)	0.0196 (8)	0.0018 (6)	−0.0026 (6)	0.0004 (6)
C5	0.0106 (6)	0.0174 (8)	0.0178 (8)	−0.0006 (5)	−0.0019 (5)	−0.0006 (6)
C6	0.0118 (6)	0.0128 (7)	0.0162 (8)	−0.0006 (5)	0.0015 (5)	−0.0007 (6)
C7	0.0100 (6)	0.0150 (7)	0.0186 (8)	0.0003 (5)	−0.0006 (5)	−0.0020 (6)
C8	0.0104 (6)	0.0133 (7)	0.0164 (8)	−0.0013 (5)	0.0011 (5)	−0.0024 (6)
C9	0.0104 (6)	0.0148 (7)	0.0198 (8)	−0.0018 (5)	0.0006 (5)	−0.0016 (6)
C10	0.0124 (7)	0.0160 (7)	0.0213 (8)	−0.0035 (6)	0.0031 (6)	−0.0040 (6)
C11	0.0179 (7)	0.0139 (7)	0.0181 (8)	−0.0019 (6)	0.0034 (6)	−0.0008 (6)
C12	0.0139 (7)	0.0128 (7)	0.0175 (8)	0.0012 (5)	0.0007 (5)	−0.0007 (6)
C13	0.0109 (7)	0.0128 (7)	0.0181 (8)	−0.0003 (5)	0.0014 (5)	−0.0024 (6)
C14	0.0270 (9)	0.0183 (8)	0.0212 (9)	0.0041 (7)	−0.0001 (7)	0.0047 (7)
C15	0.0136 (7)	0.0254 (9)	0.0230 (9)	−0.0066 (6)	0.0031 (6)	−0.0021 (7)
C16	0.0098 (6)	0.0199 (8)	0.0268 (9)	0.0020 (6)	−0.0003 (6)	0.0013 (7)
C17	0.0148 (8)	0.0326 (10)	0.0259 (9)	0.0016 (7)	0.0002 (6)	−0.0065 (8)

### Geometric parameters (Å, °)

**Table:**

O1—C1	1.3546 (17)	C8—C13	1.428 (2)
O1—H1O1	0.9454	C8—C9	1.4285 (19)
O2—C13	1.2915 (17)	C9—C10	1.363 (2)
O3—C12	1.3691 (18)	C9—H9	1.010 (18)
O3—C14	1.429 (2)	C10—C11	1.420 (2)
N1—C7	1.3129 (19)	C10—C15	1.518 (2)
N1—C6	1.4168 (19)	C11—C12	1.375 (2)
N1—H1N1	0.97 (2)	C11—H11	0.982 (19)
C1—C2	1.391 (2)	C12—C13	1.439 (2)
C1—C6	1.4015 (19)	C14—H14A	0.95 (2)
C2—C3	1.386 (2)	C14—H14B	1.01 (2)
C2—H2	0.944 (18)	C14—H14C	0.99 (2)
C3—C4	1.390 (2)	C15—C16	1.501 (2)
C3—H3	1.010 (19)	C15—H15A	0.99 (2)
C4—C5	1.384 (2)	C15—H15C	1.003 (19)
C4—H4	1.00 (2)	C16—C17	1.316 (2)
C5—C6	1.393 (2)	C16—H16	0.96 (2)
C5—H5	0.960 (19)	C17—H17A	1.01 (2)
C7—C8	1.405 (2)	C17—H17B	0.98 (2)
C7—H7	0.972 (18)		
			
C1—O1—H1O1	106.5	C8—C9—H9	115.5 (10)
C12—O3—C14	116.60 (12)	C9—C10—C11	118.66 (14)
C7—N1—C6	125.65 (13)	C9—C10—C15	121.01 (14)
C7—N1—H1N1	112.1 (13)	C11—C10—C15	120.27 (15)
C6—N1—H1N1	122.2 (13)	C12—C11—C10	121.80 (15)
O1—C1—C2	122.72 (13)	C12—C11—H11	118.7 (12)
O1—C1—C6	117.99 (13)	C10—C11—H11	119.5 (12)
C2—C1—C6	119.29 (13)	O3—C12—C11	125.46 (14)
C3—C2—C1	120.38 (14)	O3—C12—C13	113.32 (13)
C3—C2—H2	121.2 (11)	C11—C12—C13	121.22 (14)
C1—C2—H2	118.4 (11)	O2—C13—C8	122.23 (14)
C2—C3—C4	120.32 (15)	O2—C13—C12	121.86 (13)
C2—C3—H3	121.3 (11)	C8—C13—C12	115.91 (13)
C4—C3—H3	118.4 (11)	O3—C14—H14A	107.5 (12)
C5—C4—C3	119.76 (14)	O3—C14—H14B	110.9 (12)
C5—C4—H4	121.5 (12)	H14A—C14—H14B	108.0 (17)
C3—C4—H4	118.7 (12)	O3—C14—H14C	111.6 (11)
C4—C5—C6	120.37 (14)	H14A—C14—H14C	111.9 (16)
C4—C5—H5	119.9 (12)	H14B—C14—H14C	106.9 (16)
C6—C5—H5	119.7 (12)	C16—C15—C10	112.41 (13)
C5—C6—C1	119.88 (14)	C16—C15—H15A	113.0 (12)
C5—C6—N1	122.62 (13)	C10—C15—H15A	105.5 (12)
C1—C6—N1	117.48 (13)	C16—C15—H15C	109.9 (10)
N1—C7—C8	124.88 (14)	C10—C15—H15C	110.7 (10)
N1—C7—H7	114.9 (10)	H15A—C15—H15C	105.0 (16)
C8—C7—H7	120.2 (10)	C17—C16—C15	125.35 (17)
C7—C8—C13	121.01 (13)	C17—C16—H16	119.6 (12)
C7—C8—C9	117.85 (13)	C15—C16—H16	115.0 (12)
C13—C8—C9	121.15 (14)	C16—C17—H17A	121.2 (12)
C10—C9—C8	120.97 (14)	C16—C17—H17B	121.3 (12)
C10—C9—H9	123.5 (10)	H17A—C17—H17B	117.4 (17)
			
O1—C1—C2—C3	178.84 (14)	C8—C9—C10—C15	179.82 (14)
C6—C1—C2—C3	−0.3 (2)	C9—C10—C11—C12	2.0 (2)
C1—C2—C3—C4	0.3 (2)	C15—C10—C11—C12	179.24 (15)
C2—C3—C4—C5	0.4 (2)	C14—O3—C12—C11	2.1 (2)
C3—C4—C5—C6	−1.1 (2)	C14—O3—C12—C13	−178.25 (13)
C4—C5—C6—C1	1.1 (2)	C10—C11—C12—O3	−177.77 (14)
C4—C5—C6—N1	−177.01 (14)	C10—C11—C12—C13	2.6 (2)
O1—C1—C6—C5	−179.60 (14)	C7—C8—C13—O2	5.1 (2)
C2—C1—C6—C5	−0.4 (2)	C9—C8—C13—O2	−175.35 (14)
O1—C1—C6—N1	−1.4 (2)	C7—C8—C13—C12	−174.61 (14)
C2—C1—C6—N1	177.85 (13)	C9—C8—C13—C12	5.0 (2)
C7—N1—C6—C5	12.8 (2)	O3—C12—C13—O2	−5.3 (2)
C7—N1—C6—C1	−165.40 (14)	C11—C12—C13—O2	174.39 (14)
C6—N1—C7—C8	178.13 (14)	O3—C12—C13—C8	174.40 (12)
N1—C7—C8—C13	0.3 (2)	C11—C12—C13—C8	−5.9 (2)
N1—C7—C8—C9	−179.33 (14)	C9—C10—C15—C16	82.87 (19)
C7—C8—C9—C10	178.97 (14)	C11—C10—C15—C16	−94.28 (18)
C13—C8—C9—C10	−0.6 (2)	C10—C15—C16—C17	121.37 (18)
C8—C9—C10—C11	−3.0 (2)		

### Hydrogen-bond geometry (Å, °)

**Table:**

Cg1 is the centroid of the C8–C13 ring.

**Table:**

*D*—H···*A*	*D*—H	H···*A*	*D*···*A*	*D*—H···*A*
O1—H1O1···O2^i^	0.95	1.72	2.6443 (16)	166
O1—H1O1···O3^i^	0.95	2.55	3.1268 (16)	119
N1—H1N1···O2	0.97 (2)	1.85 (2)	2.6553 (18)	138 (2)
C14—H14B···Cg1^ii^	1.01 (2)	2.75 (2)	3.569 (2)	139 (2)

Symmetry codes: (i) −*x*, *y*−1/2, −*z*+1/2;  (ii) −*x*+1/2, *y*+1/2, *z*.
